# Genetic Interaction Between Two VNTRs in the SLC6A4 Gene Regulates Nicotine Dependence in Vietnamese Men

**DOI:** 10.3389/fphar.2018.01398

**Published:** 2018-12-03

**Authors:** Gea Kõks, Ele Prans, Ha Diep Thi Tran, Ngoc Bich Thi Ngo, Linh Nhat Nguyen Hoang, Hue Minh Thi Tran, Thanh Cao Ngoc, Thuoc Doan Phuoc, Xuan Dung Ho, Binh Ho Duy, Freddy Lättekivi, John Quinn, Sulev Kõks

**Affiliations:** ^1^Department of Pathophysiology, University of Tartu, Tartu, Estonia; ^2^Public Health Faculty, Danang University of Medical Technology and Pharmacy, Da Nang, Vietnam; ^3^College of Medicine and Pharmacy, Hue University, Hue, Vietnam; ^4^Molecular and Clinical Pharmacology, University of Liverpool, Liverpool, United Kingdom; ^5^Department of Reproductive Biology, Estonian University of Life Sciences, Tartu, Estonia; ^6^The Perron Institute for Neurological and Translational Science, Nedlands, WA, Australia

**Keywords:** variable number tandem repeat (VNTR), SLC6A4, smoking, nicotine dependence, fagerström test for nicotine dependence (FTND), vietnam, genetic interaction, stratification

## Abstract

Nicotine dependence is an addiction to tobacco products and a global public health concern. Association between the SLC6A4 polymorphisms and nicotine dependence is controversial. Two variable number tandem repeat (VNTR) domains, termed HTTLPR and STin2, in the SLC6A4 gene are well characterized transcriptional regulatory elements. Their polymorphism in the copy number of the repeat correlates with the particular personality and psychiatric traits. We analyzed nicotine dependence in 1,804 participants from Central Vietnam. The Fagerström Test (FTND) was used to evaluate the nicotine dependence and PCR was used to determine the SLC6A4 HTTLPR and STin2 VNTRs. The HTTLPR VNTR was associated with difficulties to refrain from smoking in a prohibiting environment. The STIn2 10/10 genotype was associated with (1) years of smoking, (2) difficulties to quit the first cigarette, and (3) higher number of cigarettes smoked per day (CPD). Stratification analysis was used to find the genetic interaction between these two VNTRs in nicotine dependence as they may synergistically regulate the SLC6A4 expression. Smokers with the S/S HTTLPR genotypes showed a much stronger association between STin2 10/10 variant and CPD. This finding is consistent with the molecular evidence for the functional interaction between HTTLPR and STin2 in cell line models, where STin2 has described as a stronger expressional regulator. Similarly, we found that STin2 is a much stronger modifier of smoking with 10/10 genotype related to higher nicotine dependence. The present study supports genetic interaction between HTTLPR and STin2 VNTRs in the regulation of nicotine dependence with the dominance of the STin2 effects. This finding could be explained by their differential effect on the SLC6A4 expression.

## Introduction

Smoking is the single major cause for health loss and premature death (Jha, 2009). Nicotine dependence is a dominant driving force that prevents successful quitting and causes a craving for tobacco. Genetic influences on nicotine dependence have been identified in several candidate gene and GWAS studies (Saccone et al., 2007; Thorgeirsson et al., 2010; Hancock et al., 2017). The most dominating associations have been found with nicotinic cholinergic receptor subunits (Saccone et al., 2009). However, development of nicotine dependence is complex, and several other genes involved in the neurotransmission are found to be associated with this process. One of the functional targets is serotonin transporter (5HTT, SERT1) also termed solute carrier family 6 member 4 (SLC6A4). This gene is a target for many psychostimulants and implicated in many psychiatric conditions and personality traits (Collier et al., 1996b; Lesch et al., 1996).

Genetic variations in several serotoninergic genes are associated with different psychiatric illnesses (Battersby et al., 1996; Collier et al., 1996a,b; Ueno, 2003). Two polymorphic variable number tandem repeats (VNTRs) in the SLC6A4 gene are amongst the most commonly analyzed polymorphisms for psychiatric and personality associations (Lesch et al., 1994; Collier et al., 1996b).

The most widely analyzed VNTR is in the promoter (termed 5HTT LPR or HTTLPR) consisting of 22–23 bp tandemly repeated units (Lesch et al., 1994; Heils et al., 1996). Initially the HTTLPR was identified as a biallelic variation comprising of 14 (short, S) and 16 (long, L) copies of the units (Haddley et al., 2012). This variation has substantial impact on the expression of the SLC6A4 with the S allele as low activity and L allele as the high activity variants (Heils et al., 1997). The functional SNP (rs25531, A/G) has been identified within the long variant giving rise to L_A_ or L_G_ variants and many other combinations between VNTR and A/G polymorphism have been determined (Nakamura et al., 2000). However, the functional relevance of L_A_ or L_G_ variants is not sufficiently understood and majority of studies consider only L or S alleles for the HTTLPR (Martin et al., 2007; Haddley et al., 2008). The low expressing S allele has been repeatedly found to be related to depression, bipolar disease, anxiety and drug addiction (Haddley et al., 2012). A second functional VNTR resides in the second intron of SLC6A4 gene and is known as STin2 (Collier et al., 1996b). It consists of 9, 10, or 12 copies of a 16–17 bp tandem sequence termed STin2.9, STin2.10, and STin2.12 (Haddley et al., 2008). Compared to the HTTLPR variations, STin2 polymorphisms are studied less frequently. However, STin2 has a positive impact on the gene expression and several reports have found its clinical relevance for disease susceptibility and drug response (Vasiliou et al., 2012; D’Souza et al., 2013). Of the two VNTRs this one has been tested functional in transgenic models and shown overlap of function to the SLC6A4 gene during development (MacKenzie and Quinn, 1999). The most prevalent alleles are 10 and 12 with shorter allele associated with lower activity of SLC6A4 (Collier et al., 1996a). STin2.10 has been found to be associated with depression, neuroticism and suicide (Lopez de Lara et al., 2007; O’Gara et al., 2008). Taken together, STin2 and HTTLPR are VNTRs regulating expression of the SLC6A4 (Haddley et al., 2012). Therefore, variations in the length of the VNTRs are not only a biomarkers for an association of a behavioral trait but can also differentially regulate the gene expression providing a mechanism partially underpinning that association.

Personality traits influence smoking behavior and higher neuroticism and anxiety has been found in smokers (Patton et al., 1997). Neuroticism is a personality trait with well-known heritable component, it correlates with anxiety, mood disorders and smoking behavior (O’Gara et al., 2008). Higher neuroticism is associated with more intensive cigarette consumption and less efficient smoking cessation (O’Gara et al., 2008).

On the other hand, smoking is shown to be involved in suicidal behavior, but this association has been a controversial for many years (Doll and Peto, 1976; Davey Smith and Munafo, 2015). One large prospective study based on 300,000 male United States Army personnel identified significantly increased relative risk for suicide among smokers (Miller et al., 2000). The risk of suicide increased with the number of cigarettes smoked per day (Miller et al., 2000) and this result has resulted in much debate of its significance (Sheikh, 2000; Smith and Phillips, 2001). Our previous study based on the Estonian National Death Registry data confirmed that smokers have significantly higher incidence for suicide as cause of death (Koks et al., 2017). Significantly increased number of suicides is an indication of the increased risk for mental disorders and self-inflicting behavior among smokers.

As VNTRs in SLC6A4 gene are related to the mood disorders, suicide risk and smoking-related personality traits, we wanted to address the association between SLC6A4 and nicotine dependence. Existing data support that variations in the SLC6A4 gene are involved in tobacco dependence and smoking behavior (Watanabe et al., 2011; de Lima et al., 2012; Pizzo de Castro et al., 2014). The present study aimed to analyse the association of two regulatory VNTRs in the SLC6A4 gene and their interaction in the nicotine dependence of Vietnamese men. Nicotine dependence was measured using the Fagerström test (FTND) that is based on six questions about different aspects of the smoking activity and describing physical nicotine dependence (Fagerstrom, 1978; Heatherton et al., 1991). FTND is widely used and accepted test for smokers with thorough validation to reflect the degree of physical dependence (Fagerstrom, 1978; Fagerstrom and Schneider, 1989). FTND has become a standard and robust approach to measure nicotine dependence for genetic studies. We found association between the STin2 polymorphism and several aspects of nicotine dependence in Asian men including a specific genotype of HTTLPR and STin2 in the nicotine dependence.

## Materials and Methods

### Study Design and Participants

A community based cross-sectional study was performed in 2015 in Da Nang and Hue, Central Vietnam. Altogether, 1,822 participants consisting of 1,453 smokers and 369 non-smokers were recruited with balanced age distribution to reduce the effect of potential confounders. Only data of men were collected due to the already demonstrated extremely low prevalence of smoking in Vietnamese women. All participants were over 18 years of age. The Ethics Review Committees on Human Research of the University of Tartu, Da Nang University of Medical Technology and Pharmacy and Hue University of Medicine and Pharmacy approved the protocols and informed consent forms used in this study. All participants signed a written informed consent form during the completion of the questionnaire.

### Smoking Data

We defined smokers as an individual that had smoked for at least one year. This definition fits with the primary goal of study to analyse nicotine dependence. Using threshold of one year would give substantial time for development of nicotine dependence. This time-based threshold would also filter out persons who occasionally tried smoking but eventually did not became dependent. Therefore, the temporal threshold excludes short-time smokers from the study and only people with some minimal regular smoking experience were included. The original questionnaire used in the study contained 19 questions and was designed to collect demographic information, smoking behavior and nicotine dependence. The collected data was then inputted into the web-based database using the RedCap software,^1^ which is now available for any other future studies (Harris et al., 2009). RedCap is an abbreviation for the Research Electronic Data CAPture and is an innovative software to support clinical and translational research. RedCap is a workflow methodology for rapid development and deployment of electronic data capture which can be used in a clinical setting. Therefore, it is an ideal platform for subsequent smoking studies.

The following questions were asked during data collection:

1. Year of birth
2. Sex
3. Ethnicity
4. “Have you ever smoked at least one year?”
5. “Are you a current or former smoker?”
6. Age of starting smoking
7. Age of stopping smoking
8. Types of tobacco products
9. The main cause of smoking
10. “How soon after waking up do you smoke your first tobacco?”
11. “Do you find it difficult to refrain from smoking in places where it is forbidden?”
12. “Which tobacco would you hate most to give up?”
13. “How many cigarettes per day (CPD) do you smoke?”
14. “Do you smoke more frequently in the morning?”
15. “Do you smoke even if you are sick in bed most of the day?”
16. “Have you ever tried to quit smoking?”
17. “For what reasons have you tried to quit smoking?”
18. “Have you re-started smoking again?”
19. “Why did you re-start smoking again?”

The first questions were used to assess the smoking habits more generally. Questions 10 to 15 are part of the Fagerström test for nicotine dependence (FTND). Responses to this section were converted to numerical values and a summary score out of ten was generated. Ten is the maximum value for the FTND and the scoring was based on the revised version of the test (Heatherton et al., 1991). The remaining questions described quitting behavior and reasons for quitting (health, price) and analyzed different motivators to restart smoking. This design for the questionnaire meant that it was short, relatively easy to use and is now imported to the web-based platform for convenient use via personal computers or mobile devices.

### Saliva Collection and DNA Extraction

DNA was collected and extracted from saliva using Norgen saliva collection, preservation and isolation kit (RU35700)^2^. Briefly, 2 ml of saliva was collected, mixed with the preservative reagent and transported to the lab in Tartu, Estonia. DNA was extracted according to the manufacturers protocol using Proteinase K digestion and alcohol precipitation.

### DNA Genotyping

Assay for genotyping the HTTLPR and STin2 is generally based on the method described by Wendland et al. (2006) with slight modifications. Multiplex polymerase chain reaction (PCR) protocol was used along with two different primer pairs (Supplementary Table S1) to amplify fragments including HTTLPR (S – 469bp and L – 512 bp) as well as STin2 VNTR genotypes (9, 10, and 12 repeats). The final reaction volume (20 ul) contained 1x AmpliTaq Gold MasterMix (Applied Biosystems, California, CA, United States) and oligonucleotide primers (TAG Copenhagen A/S) (Supplementary Table S1) at final concentration of 200 and 350 nM each, respectively. The final amount of gDNA was 25 ng. Thermal cycling consisted of 4 min of initial denaturation at 95°C followed by 41 cycles of 95°C (20 s), 62°C (20 s), and 72°C (20 s) each with a final extension step of 2 min at 72°C. Subsequently, 10 ul of PCR product was loaded onto a 2% agarose gel, run for 1 h and 10 min at 160V in TBE and visualized by ethidium bromide.

### Statistical Analysis

Statistical analysis was performed with *R studio* by using package *SNPassoc*. Package *SNPassoc* is designed to carry out most common analyses in association studies (Gonzalez et al., 2007). The package combines tools to carry out an analysis between a single SNP and phenotype (quantitative or categorical) under different genetic models: codominant, dominant, recessive, over-dominant and log-additive. The statistical significance of a given SNP is tested using likelihood ratio test, LRT. The standard output consists of samples size, genotype frequencies, effect sizes (different measures for quantitative and categorical data), *p*-value and the Akaike Information Criterion (AIC) of each genetic model (Gonzalez et al., 2007).

The phenotypes we involved in the analysis were the smoking intensity, the years of smoking, FTND and single values of each FTND question. Smoking behavior was divided into two major categories: current smokers (1,453 persons) and never smokers (369 persons). Smoking activity was measured in four categories; below 10, 11–20, 21–30, and more than 31 CPD. Based on original answers, Heaviness of Smoking Index (HSI) was also calculated (Heatherton et al., 1989). This index h has been shown to be comparable with the FTND (Chabrol et al., 2005). FTND seems to perform better than HSI in cases of low nicotine dependence (Perez-Rios et al., 2009).

## Results

### HTTLPR Polymorphism

**FIGURE 1:**
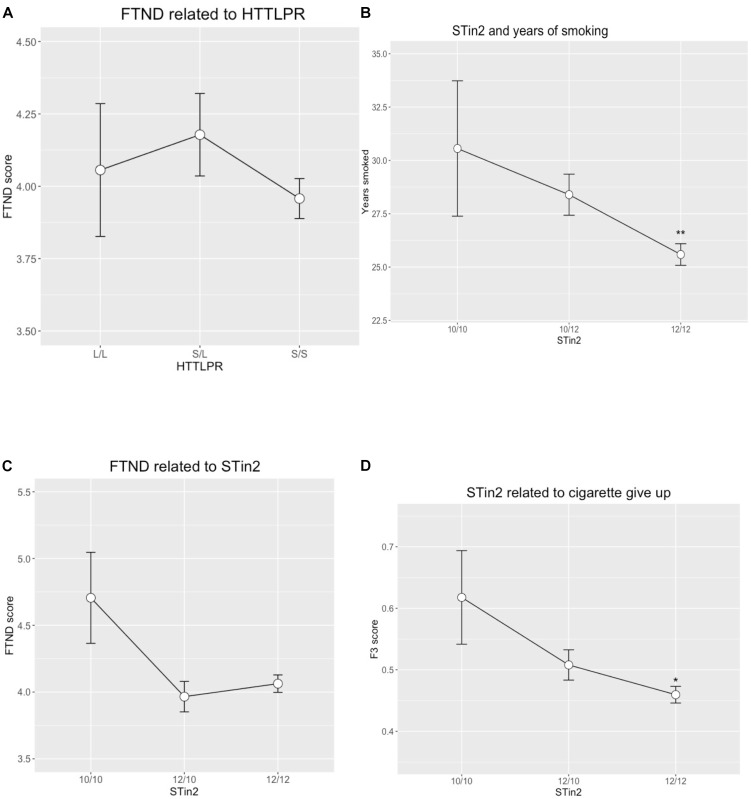
**(A)** FTND scores related to the genotypes of HTTLPR. **(B)** Years of smoking are associated with the genotypes of STin2. **(C)** Interrelations between FTND scores and STin2 genotypes. **(D)** STin2 genotypes are associated with difficulties in giving up the first cigarette a day. ^∗^p < 0.05; ^∗∗^p < 0.01.

HTTLPR genotypes departed from the Hardy-Weinberg equilibrium, but as it was caused by the loss of heterozygosity this could indicate natural variabilities in the South-Asian population (Chen et al., 2017). Smoking status (smoker versus non-smoker) was not associated with the variations in the HTTLPR locus (Table 1). FTND score (Figure 1A) and most of the questions were also not associated with the HTTLPR variations including the number of quitting attempts and HSI score. Fagerström test question 2 (F2) “Do you find it difficult to refrain from smoking in places where it is forbidden” was statistically significantly associated with the HTTLPR alleles under dominant genetic model. L allele was dominantly associated with increased difficulties to refrain smoking when it is forbidden (Supplementary Table S2). The OR was 1.32 (CI 1.02–1.70) with the *p*-value of 0.04.

**Table 1:** Results of the genotyping of two VNTRs in the SL6A4 gene, HTTLPR and STin2.

VNTRs		Never-smokers	Smokers	OR	95% CI	X^2^	*P*-value	Total	HWE *p*-value
HTTLPR	SS	235	925	1.00	NA			1160	< 0.001
	SL	73	230	1.25	0.92–1.68	2.33	0.14	303	
	LL	27	91	1.17	0.73–1.82	2.33	0.5	118	
	S	543	2080	1.00	NA			2623	
	L	127	412	1.18	0.95–1.47	2.02	0.15	539	
STin2	10/10	8	34	1.00				42	0.14
	10/12	93	320	1.22	0.57–2.94	1.48	0.61	413	
	12/12	268	1085	1.03	0.49–2.45	1.48	0.90	1353	
	10	109	388	1.11	0.89–1.40	0.72	0.40	497	
	12	629	2490	1.00	NA			3119	

*Basic statistic, Hardy-Weinberg equilibrium and association analysis with smoking status is indicated.*

### STin2 Polymorphism

The STin2 polymorphism did not depart from the Hardy-Weinberg equilibrium, but similarly to HTTLPR, was not associated with smoking status (Table 1). However, the years of smoking were under dominant (*p*-value = 0.01) and codominant (*p*-value = 0.03) models significantly associated with the STin2 variations with allele 10 being related to the longer smoking history (Supplementary Table S3). This association had a linear nature and is illustrated in the Figure 1B. The mean of the years for smoking was 25.6 years for 12/12, 28.4 years for 10/12 and 30.6 years for 10/10 genotypes (*p*-value = 0.03). STin2 allele 10/10 also had a trend to be associated with the summary score of FTND without reaching statistical significance. Study participants with genotype 10/10 had an average FTND score of 4.7 whereas participants with 10/12 or 12/12 had an average FTND score of 4.0 (Figure 1C). STin2 genotypes were significantly associated with two questions in the FTND. Responses to question 3 ”Which tobacco would you hate most to give up?” were associated with STin2 genotypes under log-additive model with OR 1.27 (CI 1.03–1.56) and *p*-value 0.02. Therefore, the persons with 10/10 genotype have difficulties in giving up the morning cigarette, and they rated morning cigarette as the most precious. This effect was distinct in the other genotypes as those with 12/12 valued it the least while persons with 10/12 genotype were intermediate (Supplementary Table S4 and Figure 1D). Among the persons with 12/12 genotype, less than half of subjects valued the morning cigarette over any other. Among the persons with 10/12 and 10/10 genotypes, more than half valued morning cigarette over any other (OR 1.27, CI 1.00–1.61, *p*-value 0.05, Figure 1D).

Question number four in FTND is ”How many CPD do you smoke?” and the answer is given in four categories, less than 10, 11–20, 21 to 30, and more than 31 CPD. According to the original FTND test, these categories were scored from 0 to 3 so that category less than 10 scored 0, 11–20 scored 1, 21–30 got 2 and more than 31 was scored with 3. STin2 genotypes were significantly associated under dominant and over-dominant model with the lowest CPD for the heterozygous persons with the 10/12 genotype (*p*-value = 0.02, Table 2 and Supplementary Figure S1). Taken together, while HTTLPR polymorphism had only modest association, STin2 polymorphism has clear evidence for the link with the nicotine dependence.

**Table 2:** Polymorphisms in the second intron (STin2) of SLC6A4 gene are associated with the F4 question in the Fagerström Test for Nicotine Dependence, “How many cigarettes per day do you smoke?” Number of cigarettes was divided into four categories (“< 11”, “11–20,” “21–30,” and “> 30”) and transformed into Fagerström score points, 0, 1, 2, and 3.

Models	Genotype	*n*	Mean	Dif.	95% CI	*P*-value	AIC
Codominant	1212	1073	0.74	0.00	NA	0.06	3259
	1012	319	0.63	−0.11	−0.21 to −0.02		
	1010	34	0.79	0.05	−0.21 to 0.31		
Dominant	1212	1073	0.74	0.00	NA	0.04	3258
	1012-1010	353	0.65	−0.10	−0.19 to −0.00		
Recessive	1212-1012	1392	0.72	0.00	NA	0.57	3262
	1010	34	0.79	0.08	−0.18 to 0.33		
Overdominant	1212-1010	1107	0.74	0.00		0.02	3257
	1012	319	0.63	−0.11	−0.21 to −0.02		
log-Additive				−0.06	−0.14 to 0.01	0.11	3260

### Interaction and Stratification Analysis

HTTLPR and STin2 are two polymorphisms which demonstrate transcriptional regulatory properties that could act combinatorially on SLC6A4 expression and genetic association studies should consider this (Ali et al., 2010). We performed the association analysis with the interaction term between these two VNTRs and did not find any significant interactions.

An alternative approach to analysing the two polymorphisms with well-established molecular interaction is stratification. The cohort was stratified by the HTTLPR or STin2 genotypes, and association testing with different genetic models reapplied. In specific cases, the effects of single-marker association results became statistically more significant after stratification.

Stratification based on the STin2 genotypes verified the association between HTTLPR variations and one’s ability to refrain from smoking when it is prohibited (Table 3). More precisely, participants with 12/12 genotypes showed significant associations under dominant (*p*-value = 0.02) and over-dominant (*p*-value = 0.02) genetic models between F2 scores and HTTLPR genotypes. The odds ratio for not to withstand smoking urge was 1.43 (CI 1.05–1.95) in persons with S/L or L/L genotypes.

**Table 3:** After stratification for the STin2 genotypes, the association of HTTLPR polymorphism with the F2 question in the Fagerström Test for Nicotine Dependence, “Do you find it difficult to refrain from smoking in places where it is forbidden?” became more significant.

Models	Genotype	“No”	“Yes”	OR	95% CI	*P*-value	AIC
Codominant	SS	437	283	1.00	NA	0.05	1267
	SL	80	79	1.52	1.08 to 2.15		
	LL	31	24	1.20	0.69 to 2.08		
Dominant	SS	437	283	1.00	NA	0.02	1265
	SL-LL	111	103	1.43	1.05 to 1.95		
Recessive	SS-SL	517	362	1.00	NA	0.72	1270
	LL	31	24	1.11	0.64 to 1.92		
Overdominant	SS-LL	468	307	1.00		0.02	1265
	SL	80	79	1.51	1.07 to 2.12		
log-Additive		548	386	1.24	0.99 to 1.55	0.06	1267

*Results of persons with the genotype 12/12 are presented.*

When HTTLPR genotypes stratified participants into L/L, S/L, and S/S groups the STin2 association with CPD became stronger in subjects with S/S genotypes (Table 4). In smokers with S/S genotype the STin2 had a significant co-dominant effect with the *p*-value 0.006. The difference described in case of STin2 alone became stronger as the persons with the S/S and 10/12 genotype have significantly lower CPD compared to the participants with 10/10 or 12/12 genotypes.

**Table 4:** After stratification for the HTTLPR genotypes, the association of STin2 polymorphism with the F4 question in the Fagerström Test for Nicotine Dependence, “How many cigarettes per day do you smoke?” became more significant. Results from stratum S/S are presented.

Models	Genotype	*n*	Mean	Dif.	95% CI	*P*-value	AIC
Codominant	1212	720	0.75	0.00	NA	0.02	2091
	1012	182	0.58	−0.17	−0.29 to −4.63e-02		
	1010	15	0.87	0.12	−0.27 to 5.01e-01		
Dominant	1212	720	0.75	0.00	NA	0.02	2091
	1012-1010	197	0.60	−0.15	−0.27 to −2.83e-02		
Recessive	1212-1012	902	0.72	0.00	NA	0.45	2096
	1010	15	0.87	0.15	−0.24 to 5.36e-01		
Overdominant	1212-1010	735	0.75	0.00		0.006	2089
	1012	182	0.58	−0.17	−0.29 to −4.89e-02		
Log-Additive				−0.11	−0.21 to −1.96e-05	0.05	2093

## Discussion

Our study was designed to analyse the potential genetic association of the two regulatory VNTRs within the SLC6A4 gene in nicotine dependence. We collected DNA from 1,435 smokers and 369 non-smokers and genotyped both VNTRs. The study population is from Central Vietnam, from Da Nang and Hue cities. FTND was utilized to provide reliable and comparable measures for physical nicotine dependence (Fagerstrom, 1978; Heatherton et al., 1991).

HTTLPR genotypes departed from the Hardy-Weinberg equilibrium, but this is most likely caused by the population substructure and not by the genotyping error. Several pieces of evidence support that reasoning, First, the genotypes of STin2 were in Hardy-Weinberg equilibrium. Second, a recent study indicated that in South-Asian populations the departure is quite common (Chen et al., 2017). Thirdly, the Hardy-Weinberg departure that is associated with a loss of heterozygosity may be explained by the natural or biological causes including population stratification (Chen et al., 2017).

The majority of the study group were smokers, only 20% of participants were non-smokers. More smokers were collected because the primary goal was to analyse the genetics of nicotine dependence and data from non-smokers was only used for smoking status comparisons. In the present study, we considered an individual as a smoker if he had smoked regularly for at least one year (Doll and Hill, 1950). This definition was introduced by Doll and Hill in their seminal paper, where they described the connection between smoking and lung cancer (Doll and Hill, 1950). This definition of a smoker was used because it was easier to understand for participants and eliminated casual smokers with very little smoking activity.

Two well-characterized regulatory VNTRs in the SLC6A4 were analyzed to evaluate the variability of the gene in smoking (Battersby et al., 1996; MacKenzie and Quinn, 1999; Haddley et al., 2012). Polymorphism in these VNTRs is also correlated with several psychiatric diseases and personality traits (Battersby et al., 1996; O’Gara et al., 2008; Mercer et al., 2012). Nicotine dependence itself is addictive behavior and is the main reason why quitting of smoking is so difficult. As smoking is related to many distinct health consequences, reducing smoking in the population is a significant public health challenge. Smokers die 10 years earlier than non-smokers on average (Koks et al., 2017). Smoking has been recognized for many years and confirmed in well characterized reproducible studies as a major cause of lung cancer and for chronic lung diseases causing premature mortality (Doll and Hill, 1999; Doll et al., 2004). However, lung diseases are not the only causes of mortality induced by smoking. Several reports have identified the association between suicide and smoking (Doll et al., 1994; Lucas et al., 2013) including dose-response relationship between cigarettes smoked per day (CPD) and suicide risk is described in smokers (Lucas et al., 2013).

While the relation between smoking and suicide is a source of debate, smoking is related to individual personalities and psychiatric disorders that are linked to suicide or self-inflicting behavior (Patton et al., 1997; Brody et al., 2005; O’Gara et al., 2008). Similarly, personality traits and mood disorders are associated with distinct genetic markers, most commonly related to serotonergic transmission. SLC6A4 is one of the most intensively studied targets in psychiatric and psychological reports. That makes SLC6A4 an exciting candidate for the genetic association studies on smoking behavior.

Our study aimed to find association between nicotine dependence and SLC6A4. We focused on well-characterized VNTRs which have not only been associated with mental health status but also their ability to act as transcriptional regulatory domains is recognized. The genotype of HTTLPR and STin2 is associated with expression of SLC6A4 and high versus low variants exist (Vasiliou et al., 2012). HTTLPR S allele is considered to be low expressing variant as is STin2 10 variant. Moreover, these VNTRs interact with each other in the regulation of SLC6A4 expression and different haplotypes (L/L 12/12 versus S/S 10/10) have different effects (Vasiliou et al., 2012; D’Souza et al., 2013).

Results from present study support the findings from existing literature and give further evidence for the involvement of SLC6A4 in nicotine dependence (O’Gara et al., 2008; Chu et al., 2009). Our present study significantly advances existing literature on the role of the SLC6A4 in nicotine dependence as it indicates differential and interactive effect of HTTLPR and STIn2 in nicotine dependence. With the promoter HTTLPR alone we did not observe strong associations in any of the questions addressed apart from under dominant models in which there was an association between the L allele and the question F2 (Supplementary Table S2). However, after stratification for the STin2 genotypes, the association of HTTLPR LL or SL genotypes with question F2 became stronger in smokers with the Stin2 12/12 genotypes. Interaction indicates that those with two combined high activity variants (12 and L) have more difficulties to refrain from smoking indicating higher nicotine dependence.

The primary genetic associations were found with the STin2 variants. Specifically the association between dependence and the low activity genotype (10/10) was found. In all three phenotypes - years of smoking, “Which tobacco would you hate most to give up” (F3 question) and ”How many CPD do you smoke?” (F4 question), the genetic models were dominant or over-dominant with main effects from the 10/10 genotypes.

Further, after stratification by the genotypes of HTTLPR, persons with S/S genotypes exhibited even stronger association between 10/10 genotype and question F4 under dominant and over-dominant models. Therefore, low activity VNTR variants interact at the genetic level increasing the association with nicotine dependence. The most plausible explanation is a mechanistic combinatorial action of these regulatory domains observed in cell line models upon gene expression. The odds ratios were more pronounced in cases with the STin2 10/10 variants than in 10/12 and 12/12 variants. This difference indicates a stronger impact of the STin2 VNTR in modulating nicotine dependence. Present study advances our knowledge about the nicotine addiction by describing the interaction between two functional VNTR loci in SLC6A4 gene in modifying the smoking behavior. This interaction is evident after stratification indicating the importance of personalized and stratified approach in the management of nicotine addiction. Our study also provides an excellent example that the interactions between different VTNR loci should be taken into consideration in genetic association studies.

The main limitation of present study is the lack of correction for multiple testing. The main reason we decided not to apply correction is the exploratory nature of the study. The significant functional interaction between the VNTRs makes correlation between these two markers very high. This means that that markers form linked haplotypes and are not inherited independently. While we also tested different genetic models for different questions, this approach is quite similar to the model fitting procedure. We also used stratification to find interaction between markers. In order to evaluate the model fitting AIC was used. Authors find that this approach is more flexible for the analytical situations where markers are highly correlated and where conservative corrections could destroy the existing associations. However, we consider the lack of corrections as a study limitation and the additional independent studies are needed to confirm this finding.

A final limitation of the study is the lack of cotinine measurements and the nicotine metabolism data. Nicotine metabolism has genetic background and is complex biochemical process. Analysis for the cotinine content would be used as proxy for the nicotine metabolism and could help to make Fagerström data more consistent. Genotyping for the nicotine metabolizing genes would be another option to take into account potential differences in the nicotine metabolism. Further studies should work with these limitations.

## Conclusion

We have analyzed the involvement of SLC6A4 genetic variations in nicotine dependence and identified significant associations of the STin2 variant with specific measures of nicotine dependence. The STin2 10/10 genotype is associated with smoking persistence (years of smoking) and difficulties in stopping smoking. This effect was stronger in those with S/S HTTLPR genotypes indicating genetic interaction between these two VNTRs in the nicotine dependence. It could indicate that those with SLC6A4 low activity genotypes (10/10 and S/S) require extra support for smoking cessation.

## Author Contributions

SK conceived the study and wrote the manuscript. GK planned the study, organized the sample collection, questionnaire, genotyping, and data analysis. EP performed the genotyping and data management. FL analyzed the data. JQ analyzed and interpreted the data, and wrote the manuscript. XH, BHD, HaT, NN, LH, HuT, TCN, and TDP helped in sample collection and answering the questionnaire.

## Conflict of Interest Statement

The authors declare that the research was conducted in the absence of any commercial or financial relationships that could be construed as a potential conflict of interest.

## Footnotes

1 https://www.project-redcap.org

2 https://norgenbiotek.com
