# Supplementary material for: Genetic Interaction Between Two VNTRs in the SLC6A4 Gene Regulates Nicotine Dependence in Vietnamese Men
Source: Front Pharmacol. 2018 Dec 3;9:1398. doi: 10.3389/fphar.2018.01398 (PMC6287221; doi:10.3389/fphar.2018.01398)
Supplement: Supplementary file 1 [file Data_Sheet_1.PDF]

Figure 1S, STin2 related to CPD

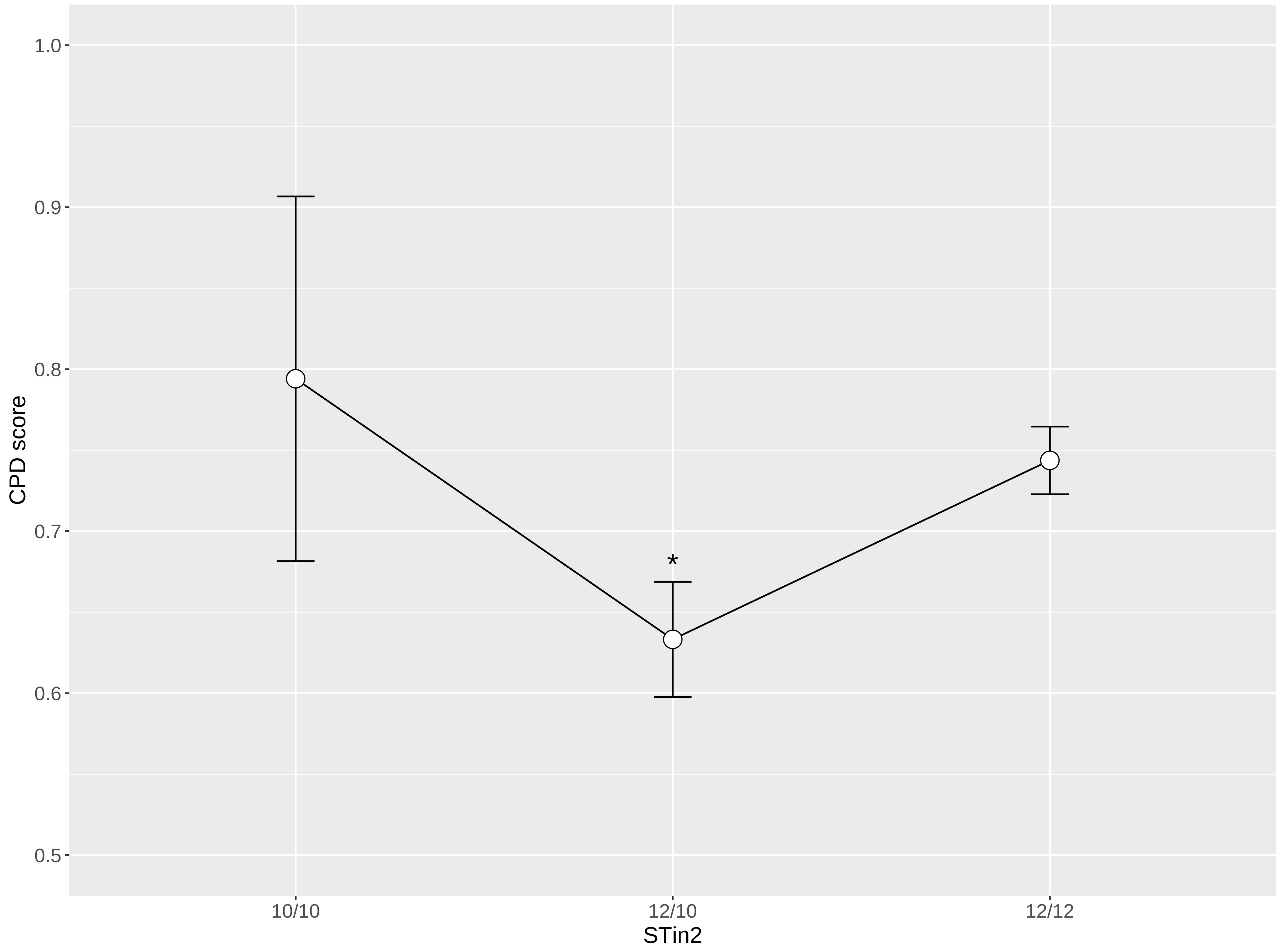

Table 1S. Oligonucleotide primer sequences and PCR product sizes used in current study.

Polymorphisms	Pirmer seqeuences		PCR product sizes
**5-HTTLPR**	Forward	5'-TCC TCC GCT TTG GCG CCT CTT CC-3'	L - 469 bp
	Reverse	5'-TGG GGG TTG CAG GGG AGA TCC TG-3'	S - 512 bp
**STin2**	Forward	5'-GGG CAA TGT CTG GCG CTT CCC CTA CAT A-3'	10 - 267 bp
	Revers	5'-TTC TGG CCT CTC AAG AGG ACC TAC AGC-3'	12 - 300 bp

Table 2S. Promoter Polymorphism in (HTTLPR) SLC6A4 gene is associated with the F2 question in the Fagerström Test for Nicotine Dependence, “Do you find it difficult to refrain from smoking in places where it is forbidden”.

Models	Genotype	“No”	“Yes”	OR	95% CI	P-value	AIC
**Codominant**	SS	555	362	1.00	NA	0.11	1672
	SL	122	106	1.33	0.99-1.78		
	LL	48	40	1.28	0.82-1.98		
**Dominant**	SS	555	362	1.00	NA	0.04	1671
	SL-LL	170	146	1.32	1.02-1.70		
**Recessive**	SS-SL	677	468	1.00	NA	0.40	1674
	LL	48	40	1.21	0.78-1.86		
**Overdominant**	SS-LL	603	402	1.00		0.07	1672
	SL	122	106	1.30	0.98-1.74		
**log-Additive**		725	508	1.2	0.99-1.44	0.06	1671

Table 3S. Polymorphisms in the second intron (STin2) of SLC6A4 gene are associated with the years of smoking.

Models	Genotype	n	mean	dif	95% CI	P-value	AIC
Codominant	1212	1084	25.59	0.00	NA	0.03	12536
	1012	320	28.39	2.80	0.45-5.16		
	1010	34	30.56	4.97	-1.48-11.41		
Dominant	1212	1084	25.59	0.00	NA	0.01	12534
	1012-1010	354	28.60	3.01	0.75-5.27		
Recessive	1212-1012	1404	26.23	0.00	NA	0.19	12539
	1010	34	30.56	4.33	-2.10-10.76		
Overdominant	1212-1010	1118	25.74	0.00		0.03	12536
	1012	320	28.39	2.65	0.31-5.00		
log-Additive				2.70	0.72-4.67	0.01	12534

Table 4S. Polymorphisms in the second intron (STin2) of SLC6A4 gene are associated with the F3 question in the Fagerström Test for Nicotine Dependence, “Which tobacco would you hate most to give up”.

Models	Genotype	“Other”	“First”	OR	95% CI	P-value	AIC
**Codominant**	1212	580	493	1.00	NA	0.07	1974
	1012	157	162	1.21	0.95-1.56		
	1010	13	21	1.90	0.94-3.83		
**Dominant**	1212	580	493	1.00	NA	0.05	1973
	1012-1010	170	183	1.27	1.00-1.61		
**Recessive**	1212-1012	737	655	1.00	NA	0.09	1974
	1010	13	21	1.82	0.90-3.66		
**Overdominant**	1212-1010	593	514	1.00		0.17	1975
	1012	157	162	1.19	0.93-1.53		
**log-Additive**		750	676	1.2	1.03-1.56	0.02	1972
